# Evidence for cryptic sex in parthenogenetic stick insects of the genus *Timema*

**DOI:** 10.1098/rspb.2023.0404

**Published:** 2023-09-20

**Authors:** Susana Freitas, Darren J. Parker, Marjorie Labédan, Zoé Dumas, Tanja Schwander

**Affiliations:** ^1^ Department of Ecology and Evolution, University of Lausanne, Lausanne, Switzerland; ^2^ School of Natural Sciences, Bangor University, Bangor, UK

**Keywords:** parthenogenesis, cryptic sex, gene flow, insects, asexuality, *Timema*

## Abstract

Obligately parthenogenetic species are expected to be short lived since the lack of sex and recombination should translate into a slower adaptation rate and increased accumulation of deleterious alleles. Some, however, are thought to have been reproducing without males for millions of years. It is not clear how these old parthenogens can escape the predicted long-term costs of parthenogenesis, but an obvious explanation is cryptic sex. In this study, we screen for signatures of cryptic sex in eight populations of four parthenogenetic species of *Timema* stick insects, some estimated to be older than 1 Myr. Low genotype diversity, homozygosity of individuals and high linkage disequilibrium (LD) unaffected by marker distances support exclusively parthenogenetic reproduction in six populations. However, in two populations (namely, of the species *Timema douglasi* and *T. monikensis*) we find strong evidence for cryptic sex, most likely mediated by rare males. These populations had comparatively high genotype diversities, lower LD, and a clear LD decay with genetic distance. Rare sex in species that are otherwise largely parthenogenetic could help explain the unusual success of parthenogenesis in the *Timema* genus and raises the question whether episodes of rare sex are in fact the simplest explanation for the persistence of many old parthenogens in nature.

## 1. Introduction

Although sex is the most prevalent mode of reproduction in animals, many species are able to reproduce via female-producing parthenogenesis. These species can reproduce without males, either exclusively (obligate parthenogens) or facultatively (facultative and cyclical parthenogens) [1]. Parthenogenesis is associated with considerable short-term advantages relative to sexual reproduction, the two main ones being reproductive assurance (since parthenogenetic organisms only require one individual for reproduction) [2] and demographic advantage (since parthenogenic reproduction produces only females) [3,4]. However, these short-term advantages are predicted to be coupled with long-term costs, including the accumulation of deleterious alleles [5,6], and a slower rate of adaptive evolution [7,8]. These long-term costs are believed to result in the eventual extinction of obligately parthenogenetic species [4,6].

Despite the expected long-term costs of parthenogenesis, several parthenogenetic species are thought to have been reproducing solely parthenogenetically for millions of years [9,10]. This raises the question of how these old parthenogens have managed to escape the predicted long-term consequences of parthenogenesis thus far. One possible explanation would be that besides reproducing parthenogenetically, they occasionally reproduce sexually (cryptic sex). Indeed, bouts of rare sex in mainly parthenogenetically reproducing populations would be sufficient to overcome many of the long-term costs of parthenogenesis and could prevent the mutational deterioration of otherwise clonal lineages [11–13]. Consistent with this idea, several species originally thought to be obligately parthenogenetic have been recently shown to be able to exchange genetic material between individuals (cryptic gene flow), including the brine shrimp *Artemia parthenogenetica* [14], and the bdelloid rotifers *Adineta vaga* [15] and *Macrotrachella quadricornifera* [16,17], questioning the existence of ancient asexuality (see also [18]).

In bdelloid rotifers, males have never been observed [19], however patterns of allele sharing between individuals are compatible with some form of rare or non-canonical sex [15–17]. On the other hand, in *Artemia* brine shrimps it has been demonstrated that cryptic gene flow can be mediated by rare males [14]. Similar to *Artemia*, rare males are documented in many obligately parthenogenetic species [20–23], but they typically do not appear to reproduce sexually with parthenogenetic females (reviewed in [24]). In some species, males produced by parthenogenetic females can mate with females of related sexual strains, but while such matings can result in new parthenogenetic lineages via contagious parthenogenesis [25,26], they do not mediate gene flow within parthenogenetic lineages. Nevertheless, formal tests of cryptic gene flow within parthenogenetic lineages remain scarce, and rare males may therefore mediate cryptic gene flow in a broader panel of ‘obligately' parthenogenetic species than generally assumed.

In this study, we assess whether cryptic gene flow occurs in parthenogenetic species of the stick insect genus *Timema* (Phasmatodea). This genus comprises five described parthenogenetic species [27–29] which represent at least seven independently derived parthenogenetic lineages [30]. These lineages vary in age, and the oldest parthenogenetic *Timema* is thought to have been reproducing via parthenogenesis for over 1 Myr [30], making them an ‘old' asexual species. Parthenogenetic *Timema* are very homozygous as they most likely reproduce via a form of automictic parthenogenesis where heterozygosity is completely lost each generation [31,32]. Rare males have been found in four of the five described parthenogenetic *Timema* species [28,29,33], and these rare ‘parthenogenetic' males present normal male reproductive organs, and can mate and produce offspring with sexual sister-species females, although with reduced fertility [33]. It is not clear how such males are produced; however, they are thought to originate from the accidental loss of an X chromosome (aneuploidy) during parthenogenesis [33]. Given the XX/X0 mechanism of sex determination in *Timema* [34], if an XX parthenogenetic egg loses one X it could potentially develop into a male, a mechanism documented in other parthenogenetic insect species [35,36].

To test for cryptic sex in *Timema*, we use RADseq genotyping to estimate linkage disequilibrium (LD) in eight populations of parthenogenetic species. A population specific approach is appropriate for studies with these species, as they live in patchy habitats and have very limited dispersal [37]. LD can serve as a measure of genetic exchange as with strict, long-term parthenogenesis alleles will be transmitted in a single block. Note that many forms of automictic parthenogenesis involve some level of recombination and are associated with a rapid loss of heterozygosity after the inception of parthenogenesis in a sexual ancestor [38]. Under strict, long-term parthenogens, such recombination does however not affect population LD as heterozygosity can no longer decrease, either because it has eroded completely or because remaining heterozygosity tracts are mechanistically or selectively constrained [39,40]. In such cases, LD will only be broken by mutation or allele conversion, and is thus expected to be high. Sexual events on the other hand, will decrease LD, even when they are very rare. While we find no evidence for cryptic sex in six populations, we show that in two of the eight studied populations of parthenogenetic *Timema*, LD within and between chromosomes is very low, and there is a consistent LD decay along the chromosomes. In one of these populations, we also find several individuals with unusually high heterozygosity, indicative of recent sexual events. The results presented here are the first evidence for cryptic sex, most likely mediated by rare males, in ‘obligately' parthenogenetic *Timema* and contribute to the growing evidence that rare or non-canonical sex might have to be considered when studying the long-term persistence of (mostly) parthenogenetically reproducing species.

## 2. Material and methods

### (a) Samples and sequencing

We looked for evidence of gene flow in two populations from each of four parthenogenetic species of *Timema* (*T. genevievae*, *T. shepardi*, *T. monikensis*, *T. douglasi*), in a total of eight populations collected between 2010 and 2018. A population of the sexual species *T. cristinae* was also included to establish a sexual reference for LD and heterozygosity patterns based on RADseq genotypes. We used a population specific approach because *Timema* are wingless, and it has been estimated that they can travel a maximum of 128 m per generation [37], which is much less than the distance between the two studied populations in each parthenogenetic species (6, 31, 43 and 219 km apart on a straight line for *T. monikensis*, *T. shepardi*, *T. douglasi* and *T. genevievae*, respectively).

We genotyped 24 females for each of the eight parthenogenetic populations (192 females in total) and 24 individuals (12 females and 12 males) for the sexual population (see also electronic supplementary material, table S1). Additionally, because we found evidence for recent gene flow in one population of *T. monikensis* (see below) we included additional samples of *T. monikensis* from different collection years for both populations under study, which included four males from the FS population (electronic supplementary material, table S1).

DNA was extracted from the head or legs (electronic supplementary material, table S1) using the Qiagen DNeasy Blood and Tissue Kit, following the manufacturer's instructions. ddRAD libraries were prepared following the protocol from [41], with the enzymes EcoRI and MseI, and a 200–450 bp size selection after addition of Illumina TruSeq indexes. For logistical reasons, a few samples underwent a different size selection (300–500 bp). Ninety-six samples were pooled in each library thanks to barcoded adapters (see protocol in [41]). Libraries were multiplexed by pairs on two Illumina lanes using Illumina TruSeq indexes iA06 or iA12 and 150 bp single-end sequencing was performed using Illumina Hiseq 2500 at the Lausanne Genomic Technologies Facility. Reads are available under BioProject accession number PRJNA798556.

### (b) Reads processing

All scripts used in this pipeline are available online (https://github.com/SusanaNFreitas/cryptic_gene_flow). We used the STACKS software (version 2.5) [42] to de-multiplex the reads with the ‘process radtags' module from the STACKS suite. Reads were trimmed from adaptors and filtered for minimum length (80 bp) with Cutadapt 2.3 [43] and aligned against the corresponding reference genomes [31] with BWA. Sam files were sorted and converted to bam using samtools 1.4 excluding multiple alignments and PHRED quality score of 30 or less. FreeBayes (v. 1.3.3) [44] was used to call SNPs, which were then filtered by read depth (with GATK, v. 4.2.0.0 [45], keeping only positions with DP > 8 and DP < 200), genotype quality (using a custom python script, keeping SNP genotypes with QUAL > 30) and missing data (with vcftools [46], only positions with 75% data kept). The *vcfallelicprimitives* function of vcflib [47] was used to break MNPs in the vcf file into SNPs, and only biallelic SNPs were kept (eliminating indels and multi-allelic positions with custom unix oneliners). Low coverage samples were excluded from further analyses (electronic supplementary material, table S1).

### (c) Population genetics

From the RADseq reads mapped to each species' genome, we recovered 25 777, 17 997, 14 326 and 10 617 SNPs for *T. genevievae*, *T. monikensis*, *T. douglasi* and *T. shepardi*, respectively. These were then used to infer the number of clones in our populations by estimating pairwise genetic distances between individuals, visualized as neighbour-joining networks (figure 2). To explicitly look for cryptic sex, we then measured LD and LD decay in each of the eight parthenogenetic populations (figures 3–5; and electronic supplementary material, figures S2–S9) and estimated relative heterozygosity for each individual (figure 6).

The total number of SNPs per species was estimated with the R package ADEGENET 2.1.3 [48,49]. Genetic distances were estimated using the Euclidean distance with the dist() function. LD was estimated with plink 1.9 [50], the *r*^2^ value was estimated within and between linkage groups with options --*r2* and --*inter-chr*, and LD decay within linkage group with options --*r2, --ld-window 999999* and *--ld-window-kb* 8000. Only SNPs with minor allele frequency >0.2 were used to estimate *r*^2^. For LD decay, we only used SNPs with available chromosomal coordinates [31,51], which were based on chromosomal information from the species *T. cristinae* (assembly v1.3 [52]). The best-fit line estimated on the LD decay plots was generated with the geom_smooth() function using the gam method, or the LOESS method whenever fewer than 1000 data points were available (chromosomal interactions). The best-fit line was only estimated for chromosomes with more than 100 observations points. Because the X chromosome was misassembled in the v1.3 *T. cristinae* assembly, we used the positional information for SNPs on all chromosomes except the X [31,51]. *r*^2^ values per population were plotted with the package ggplot2 [53] in R. In the plots of the chromosomal LD, the black dot represents the mean *r*^2^ per chromosome. Ninety-five per cent confidence intervals were approximated using the standard error (s.e. = (standard deviation)/(square root of the sample size)), adding (for the upper limit) or subtracting (for the lower limit) to the mean twice the respective s.e. Relative heterozygosity was estimated for each individual by dividing the number of heterozygous positions by the total number of called polymorphic positions.

## 3. Results

### (a) Genetic diversity patterns: sexual versus parthenogenetic

**Figure 1.**
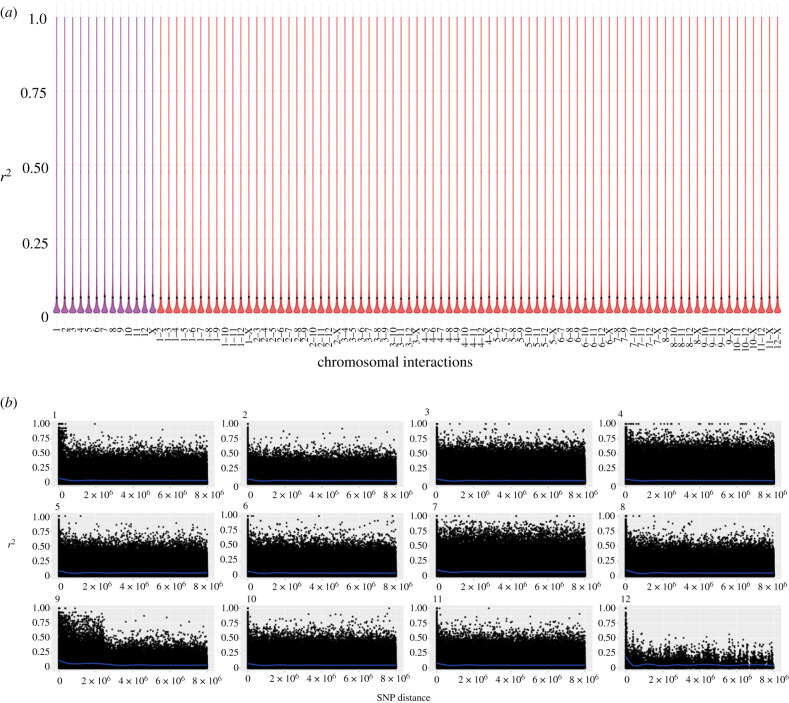
LD in the sexual species *T. cristinae*. (*a*) Violin plots of the LD values within (purple) and between (red) chromosomes (linkage groups). Black dot represents the mean LD (*r*^2^) and the black bar the approximate 95% confidence interval. (*b*) LD decay for each linkage group. LD decay was estimated for all chromosomes excluding the X, for which we did not have positional information (see Material and methods). Lines of best fit (produced using the gam method) are shown in blue.

Sexual *Timema* populations, as exemplified by the *T. cristinae* population included here, present high relative heterozygosity and genotype diversity (electronic supplementary material, figure S1, see also [31]). As expected for a sexual population, where recombination and segregation of chromosomes break LD, *r*^2^ values both within and between chromosomes were very low (figure 1*a*), even between SNPs separated by small distances (figure 1*b*).

In contrast to the sexual population with high genotype diversity, populations reproducing solely by parthenogenesis are prone to undergo recurrent sweeps. This should result in the fixation of one or few clones (genotypes), with minor genetic differences between individuals within clonal lineages. In *Timema*, strict parthenogenesis would further be associated with very low heterozygosity for all individuals in a population, since their parthenogenesis mechanism leads to the complete or largely complete loss of heterozygosity every generation [31,32]. On the other hand, if parthenogenetic *Timema* populations are undergoing rare sex we would expect to see an increase in the genotypic diversity, and perhaps some individuals with elevated heterozygosity, which would indicate that they were produced via sex.

**Figure 2.**
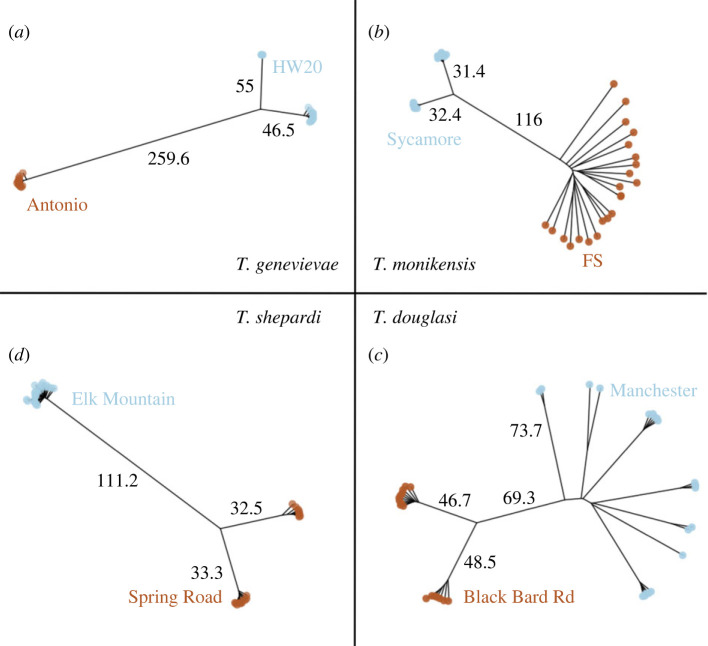
Neighbour-joining networks of pairwise distances between individuals within parthenogenetic *Timema* species: (*a*) *T. genevievae,* (*b*) *T. monikensis,* (*c*) *T. douglasi* and (*d*) *T. shepardi*. Individuals of the two studied populations in each species are distinguished by brown and blue labels.

**Figure 3.**
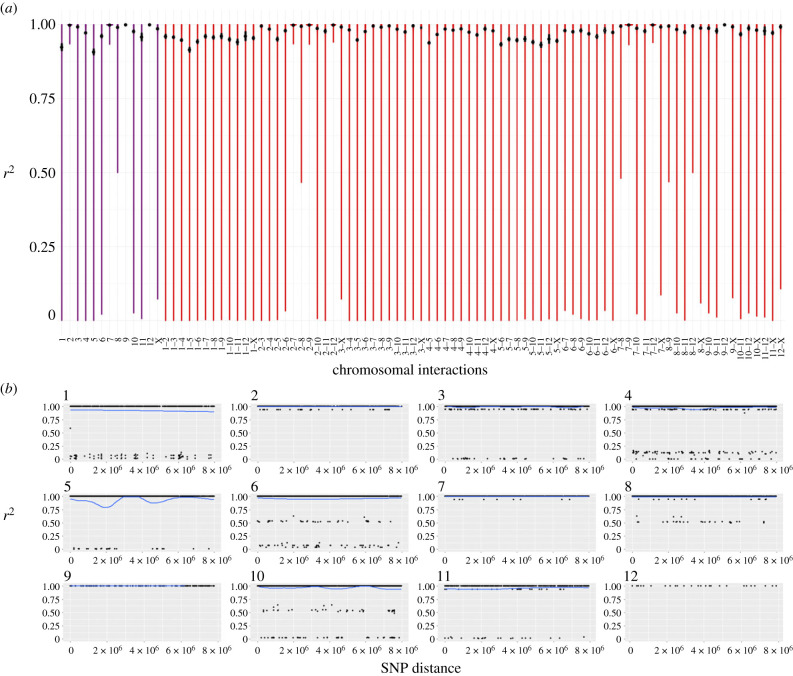
LD in the parthenogenetic species *T. genevievae* (population HW20). (*a*) Violin plots of the LD values within (purple) and between (red) chromosomes (linkage groups). Black dot represents the mean LD (*r*^2^) and the black line the approximate 95% confidence interval. (*b*) LD decay for each linkage group. LD decay was estimated for all chromosomes excluding the X, for which we did not have positional information (see Material and methods). Lines of best fit are shown in blue made using the gam method (chromosomes 1–8 and 10) and LOESS (chromosomes 9, 11). The line of best fit was only estimated when at least 100 datapoints were available. LD values for *T. genevievae*—population HW20 are presented here as an illustrative example but see electronic supplementary material, figures S2–S9 for all parthenogenetic populations.

**Figure 4.**
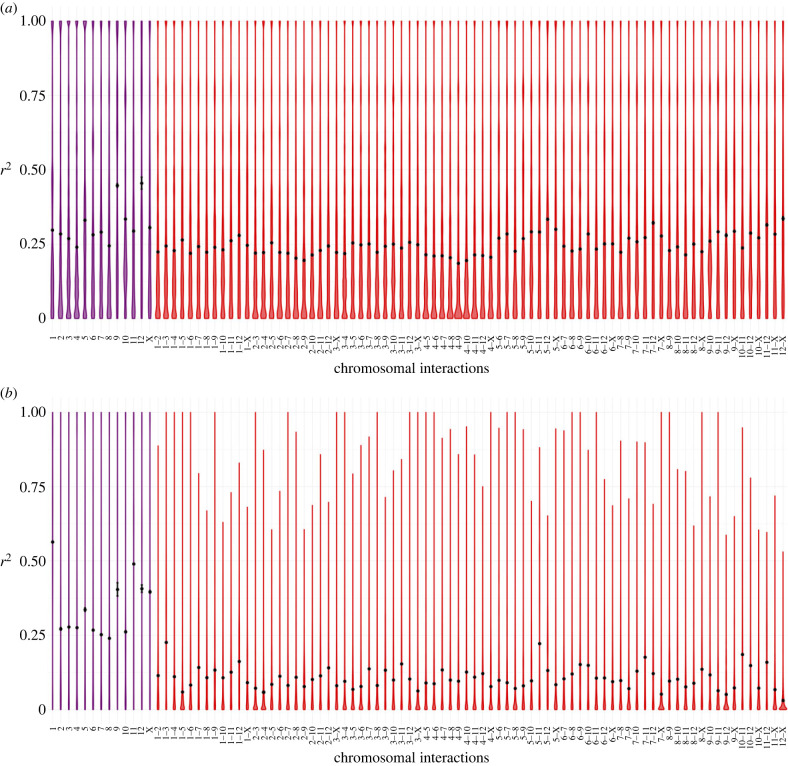
LD values in parthenogenetic populations showing evidence for gene flow. LD values within (purple) and between (red) chromosomes (linkage groups) represented in violin plots, with the black dot representing the mean LD (*r*^2^) and the black bar the approximate 95% confidence interval. (*a*) *T. douglasi*, population Manchester. (*b*) *T. monikensis*, population FS.

**Figure 5.**
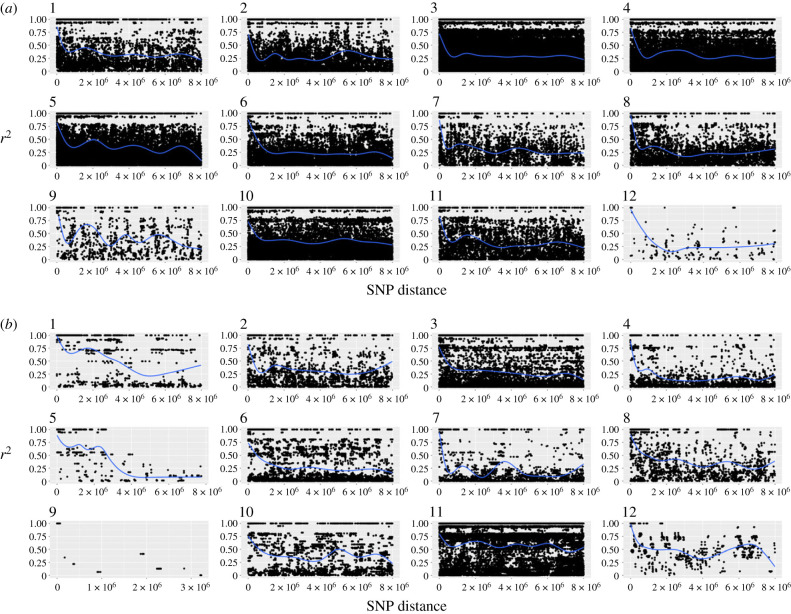
LD decay per chromosome in parthenogenetic populations showing evidence for gene flow. LD decay was estimated for all chromosomes excluding the X, for which we did not have positional information (see Material and methods). Lines of best fit are shown in blue. (*a*) *T. douglasi*, population Manchester. Lines of best fit were estimated with the gam method for all chromosomes except for chromosome 12 for which the LOESS method was used. (*b*) *T. monikensis*, population FS. Lines of best fit were estimated with the gam method for all chromosomes except for chromosomes 1, 5 and 12 for which the LOESS method was used.

We found two distinct patterns in the genetic diversity estimates (pairwise distances, LD and heterozygosity) among the populations of the parthenogenetic species. In six of the eight populations, we found no evidence for cryptic sex, as the observed patterns clearly matched the expectations for a purely parthenogenetic population: genotype diversities were very low (one or two clones per population; figure 2), and LD was high (figure 3*a*; electronic supplementary material, figures S2–3, S4-A and S5-A), and did not decay over increasing genetic distances (electronic supplementary material, figures S6–S7, S8-A and S9-A). We found clear evidence for gene flow in the remaining two populations, with high diversity of genotypes (figure 2), low LD (figure 4) and evidence of LD decay (figure 5).

### (b) Populations with no evidence for sex

**Figure 6.**
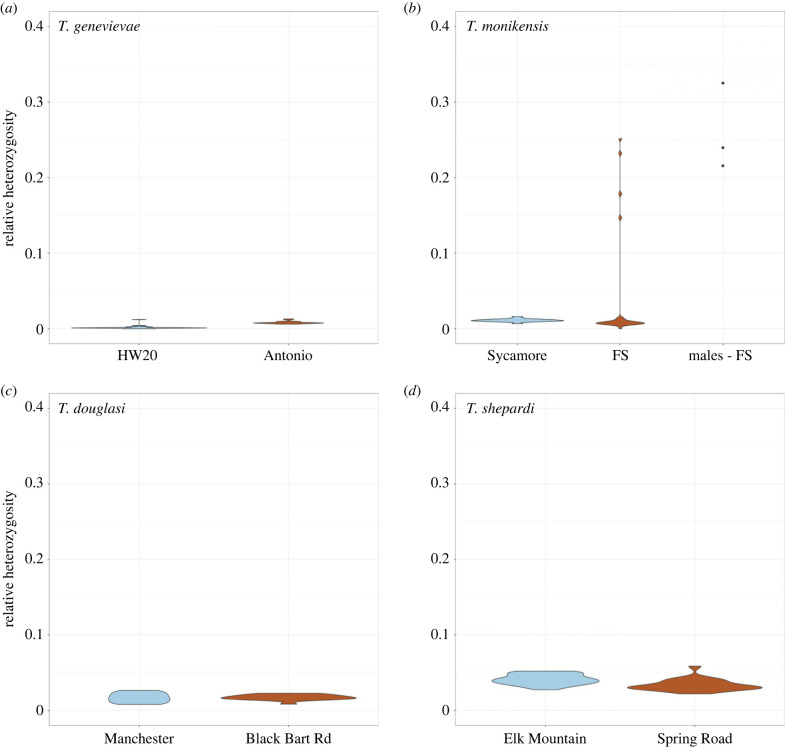
Relative heterozygosity per individual in each of the eight studied populations. (*a*) *T. genevievae*, (*b*) *T. monikensis*, (*a*) *T. douglasi*, and (*d*) *T. shepardi*. On the *x*-axis are the populations for the four parthenogenetic species studied, plus the males in the *T. monikensis*—FS population. On the *y*-axis is the relative heterozygosity estimated as the median between all chromosomes.

The six populations that presented no evidence for cryptic sex (hereafter obligately parthenogenetic populations), had either one clonal lineage, namely *T. genevievae—*Antonio and *T. shepardi—*Elk Mountain, or two clonal lineages, *T. genevievae—*HW20, *T. shepardi—*Spring Road, *T. douglasi*—Black Bart Rd and *T. monikensis—*Sycamore (figure 2). Even though we estimated LD values for all cases (electronic supplementary material, figures S2–S9), estimates for populations with only a single clonal lineage are not informative given the extremely low polymorphism they present. Nevertheless, the presence of a single clone in those populations is in itself consistent with the expectation for the (at least short-term) absence of sex. For the four populations with two clonal lineages, LD values within and between chromosomes were consistently very high (figure 3*a*; electronic supplementary material, figures S2–S5), indicative of a tight linkage between polymorphic positions. There was also no evidence of LD decay (figure 3*b*; electronic supplementary material, figures S6–S9), consistent with linkage between polymorphic positions and the absence of recombination. Finally, individuals from all six obligately parthenogenetic populations (including the two for which LD estimates are not informative due to low polymorphism) presented consistently low heterozygosity (figure 6).

### (c) Populations with evidence for sex

The patterns in the remaining two populations (*T. douglasi—*Manchester, and *T. monikensis—*FS) are, however, much different and best explained by (rare) sexual reproduction between parthenogenetic individuals. Both populations present a high diversity of genotypes, with eight different clonal lineages in *T. douglasi—*Manchester, but no clonal lineages and only independent genotypes (21) in *T. monikensis—*FS (figure 2). Regarding LD within and between chromosomes, both populations presented much lower values of *r*^2^ when compared with the strictly parthenogenetic populations (figure 3; electronic supplementary material, figures S2–S5), yet values were still elevated relative to the sexual *T. cristinae* population (figure 1). LD decay is also apparent in these two populations, with estimates consistently decreasing with increasing distance between markers (figure 5). Furthermore, for *T. monikensis—*FS, *r*^2^ values within linkage groups were higher than *r*^2^ values between different chromosomes (figure 4*b*), suggesting that segregation contributes more strongly to the high genotype diversity in this population than recombination.

### (d) Sexually produced individuals

Given the evidence for cryptic gene flow in the *T. douglasi—*Manchester and *T. monikensis—*FS populations, we investigated whether we could identify sexually produced individuals. Parthenogenetically produced *Timema* individuals are largely or completely homozygous [31,32]. By contrast, sex between different clones would manifest as elevated heterozygosity in the resulting offspring. In other words, the presence of individuals with elevated heterozygosity would indicate events of sex in the previous generation.

As expected, the relative heterozygosity values for the individuals in the six fully parthenogenetic populations were all very low, varying between 0 and 0.05. By contrast, four out of the 21 *T. monikensis* females from the population FS showed elevated relative heterozygosity (figure 6), corroborating our previous conclusions of cryptic sex in this population. The heterozygosity levels of these four individuals are as expected if these individuals were produced from crosses between individuals with low heterozygosity from the same population (electronic supplementary material, figure S10). On the other hand, none of the 20 *T. douglasi* females from the Manchester population had elevated heterozygosity, indicating that none of these females were produced sexually.

Finally, to investigate whether gene flow in *T. monikensis—*FS is mediated by rare males, and to assess whether these males are produced sexually or stem from accidental X chromosome losses, we also estimated relative heterozygosity for the four available males from that population (collected in a different year). If produced sexually, males would present elevated heterozygosity, similar to the four heterozygous females, but if resultant from X loss, males would present low heterozygosity levels. All four males were heterozygotic outliers, similar to the four highly heterozygous females (figure 6) indicating they were likely produced via sex.

### (e) Additional analyses

To corroborate our conclusions of cryptic gene flow in the *T. douglasi—*Manchester and the *T. monikensis—*FS populations we performed three additional complementary analyses. First, we found no evidence that highly heterozygous individuals were triploid, as would be expected from the rare fertilization of parthenogenetically produced diploid eggs (electronic supplementary material, figure S11). Second, we tested if our LD findings in the *T. monikensis—*FS population were driven by the heterozygous outliers. To do this, we repeated our LD analyses with heterozygous outlier individuals removed and found that LD and LD decay remained very similar (electronic supplementary material, figure S12). Finally, to test whether LD and heterozygosity in *T. monikensis* populations (both Sycamore and FS) were consistent between different years, we analysed additional individuals from previous collection years (namely, 2013 and 2015). We found similar results to our main analyses: no evidence for sex in Sycamore, and strong evidence for cryptic sex in the FS population from low LD (electronic supplementary material, figure S13), evident LD decay (electronic supplementary material, figure S14) and heterozygotic outlier individuals (electronic supplementary material, figure S15).

## 4. Discussion

While obligately parthenogenetic lineages are expected to be short lived, many are thought to have been able to persist in the long term, sometimes even for millions of years ([9,10], but see [18]). It is still unknown how parthenogenetic lineages manage to persist for so long and evade the costs of lacking sex. However, rare events of sexual reproduction have been suggested as an explanation for the persistence of long lived parthenogens, and evidence for rare gene flow in ‘obligately' parthenogenetic species is accumulating [14–17].

Rare events of sex in obligately parthenogenetic species can be difficult to demonstrate, since unless we find direct evidence (such as direct observations of sexual offspring in broods of parthenogenetic females), we need to rely on indirect evidence. Indirect evidence may include the presence of functional males, unexpectedly high genetic diversity in parthenogenetic populations or signatures of recombination (e.g. recombining haplotypes, low LD). Here, we searched for signatures of recombination and sex in parthenogenetic species of the genus *Timema* by estimating genotype diversity within populations, LD, LD decay with increasing marker distance and heterozygosity. Some forms of parthenogenesis can involve recombination and segregation [38,54], but in homozygous parthenogens such as *Timema* [31,32]*,* these two mechanisms have little to no effect on genotype diversity. In such homozygous parthenogens, signatures of recombination can therefore provide very strong evidence for sexual reproduction.

While we found no evidence for cryptic sex in six populations, we detected signatures of recombination and cryptic sex in two populations of parthenogenetic *Timema*, the population Manchester of *T. douglasi* and the population FS of *T. monikensis.* Cryptic sex, at least in *T. monikensis*, is almost certainly mediated by rare males, known to occur in *Timema* parthenogens. Three lines of evidence indicate that cryptic sex within parthenogenetic populations is a much more likely explanation for our findings than ‘contagious parthenogenesis', where males produced by parthenogenetic lineages mate with sexual females and thereby generate new parthenogenetic strains via introgression. First, sexual and parthenogenetic sister species do not overlap geographically in *Timema*, with the closest known populations being separated by hundreds of kilometres [55]. Second, sexual and parthenogenetic populations are genetically diverged [31], such that introgression would generate genetic distances beyond the intra-population distances we describe (figure 2). Finally, the heterozygosity of the putatively sexually produced individuals fit the expected heterozygosity modelled for sex within populations (electronic supplementary material, figure S10).

The uncovered patterns in the populations with signatures for cryptic sex, Manchester and FS populations, reflected different scenarios. Regarding the Manchester population (*T. douglasi*), LD within and between chromosomes was generally low (and similar), and a clear pattern of LD decay was evident across all linkage groups. Genotype diversity was relatively high (i.e. we found eight different ‘clones' among the 20 genotyped individuals), and clones were often represented by multiple individuals (one to five per clone). Finally, the heterozygosity of all females was as low as in the strictly parthenogenetic populations, indicating they were produced via parthenogenesis. These findings are best explained by rare sexual events that occurred in the past, leading to a high diversity of genotypes, but also allowing for the spread of these genotypes into clonal lineages.

The second population with evidence for gene flow, the FS population of *T. monikensis,* likely features more, and more recent sexual events than the Manchester population of *T. douglasi*. More sexual events could result via a more frequent production of males, higher propensity of females to reproduce sexually when mated, and/or for longer periods of time, for instance. The 21 genotyped females represented 21 distinct genotypes, as would be expected for a sexual but not for a largely parthenogenetic population. Consistent with the high genotype diversity, LD decay was evident for all chromosomes and LD values were generally very low. Finally, four out of the 21 females (approx. 19%) were characterized by elevated heterozygosity, and were most likely produced from a sexual cross between different genotypes (electronic supplementary material, figure S6). *Timema* parthenogenetic females were previously screened for their ability to fertilize eggs by using crosses with males from related sexual species (*T. cristinae* males for *T. monikensis* females of the FS population), but none of the 322 genotyped hatchlings were sexually produced [33]. Even though the absence of sexually produced offspring in those crosses could partly stem from species divergence and associated incompatibilities, the reciprocal crosses (*T. cristinae* females mated to *T. monikensis* males) result in large numbers of hybrid offspring [33]. Given our new results indicating *T. monikensis* are capable of reproducing sexually, this suggests that sexually produced offspring were too rare to be detected in the analysed sample of hatchlings, yet we here report that as many as 19% of the adult females of the FS population were sexually produced. One possible explanation for such a frequency difference could be that sexually produced females have a survival advantage over parthenogenetically produced ones. If that is the case, then the maintenance of mostly parthenogenetic reproduction in the FS population in spite of this apparent selection for sex will be an interesting focus for future research.

Even though we do find evidence for genetic exchange in two parthenogenetic populations, most of the populations analysed here are *de facto* parthenogenetic, and do not present any evidence of gene flow at present or in the recent past. Two scenarios could potentially explain this patchy occurrence of sex. In the first scenario, a parthenogenetic all-female population can produce males spontaneously (via e.g. X aneuploidies), and parthenogenetic females are then able to mate and produce at least some sexual offspring. Such sexual offspring would comprise males, which would then allow for the propagation of males in the population. In the second scenario, males and sexual reproduction were originally present in all populations since the split with the closest sexual ancestor and were gradually lost or even extinct in the majority of recent parthenogenetic populations. In this scenario, populations that have males at present have somehow maintained rare sex and sexually produced offspring (and thus males) perhaps through selection. In both scenarios, *de facto* parthenogenetic populations have been reproducing asexually for the last generations, but we cannot rule out these populations have not undergone episodes of (rare) sexual reproduction in the distant past.

Why sex occurs in some populations but not others is unclear and may also be a dynamic process. For example, if sex can emerge spontaneously in a population it may persist for multiple generations before going extinct due to the short-term advantages of parthenogenesis. In species that inhabit fire-prone habitats, such as *Timema,* an ecologically important short-term advantage of parthenogenesis is the ability to rapidly recolonize areas following fire with a single (female) individual. As such, the recurrent selection for parthenogenesis following fire, combined with the inherent stochasticity of extinction–recolonization dynamics of fire-prone habitats, may drive a patchy distribution of sex in *Timema* parthenogenetic populations.

As mentioned above, males are known in four out of the five described parthenogenetic species of *Timema*, including *T. monikensis* [27–29,33]. Our findings of cryptic sex in one population of *T. monikensis* now raise the question of whether males in other *Timema* species could also mediate cryptic sex in some populations, albeit much more rarely than in the FS population of *T. monikensis*. In combination with other recent reports of gene flow in parthenogenetic animal species [14–17], our findings suggest that episodes of rare sex in largely parthenogenetic species may occur more frequently than generally appreciated. Thus, it would be useful to screen additional parthenogenetic species for signatures of sex, especially species with only suggestive evidence that sexual reproduction may still be ongoing, such as parthenogenetic species with functional males, or unexpectedly high levels of genetic diversity. Males have been found in several parthenogenetic species (e.g. [20–23]), and many examples exist for high genetic diversity in parthenogenetically reproducing populations. Both these findings have been used to suggest cryptic gene flow in parthenogenetic species [56,57], but neither are reliable evidence for sex. The presence of males itself may not necessarily mean that those males can mate with parthenogenetic females, since traits required for sexual reproduction are typically vestigialized in parthenogenetic females [24]. Furthermore, males can be dysfunctional, especially in older parthenogenetic lineages where mutations may have accumulated in pathways for exclusively male functions [24]. Males produced by parthenogenetic females could also mate with sexual females, such as in the cases of contagious parthenogenesis [22,58]. Accordingly, high genetic diversity in parthenogenetic species could be explained by multiple transitions to parthenogenesis [59–61] and not indicate cryptic sex. However, future studies of such species could reveal cryptic sex in at least some of them. Finally, despite the evidence for cryptic sex in some parthenogenetic species, evidence for long-term exclusive parthenogenesis in eukaryotes has also been reported for protozoans [62] and oribatid mites [63]. Since both these groups are characterised by extremely large population sizes, these findings could be an indication of support for theory showing diminishing benefits of sex with large population size [6,64].

In conclusion, independently of how many additional parthenogenetic species will be discovered to reproduce sexually (on rare occasions), the fact that at least some presumed obligate parthenogens can reproduce sexually questions our understanding about the plasticity of sexual and parthenogenetic reproduction modes in animals. When classifying a species as sexual, we expect that all offspring are produced by sex, yet many species are capable of spontaneous parthenogenesis [54]. Accordingly, when classifying a species as obligately parthenogenetic we rarely consider occasional and/or non-canonical sex. While most reproductive systems in animals fit into a bimodal pattern of ‘sexual' or ‘parthenogenetic', we also concur with [14], that reproductive systems may contain more variation than previously assumed, and believe this could in many ways explain how old parthenogens escaped the long-term costs of the ‘lack of sex'.

## Data Availability

Raw sequence reads have been deposited in NCBI's sequence read archive under the bioproject: PRJNA798556. Scripts for the analyses in this paper are available at: https://doi.org/10.5281/zenodo.8014163 [65]. Supplementary material is available online [66].
